# Preparation of Textured Surfaces on Aluminum-Alloy Substrates

**DOI:** 10.3390/ma12010109

**Published:** 2018-12-31

**Authors:** Markéta Kadlečková, Antonín Minařík, Petr Smolka, Aleš Mráček, Erik Wrzecionko, Libor Novák, Lenka Musilová, Radek Gajdošík

**Affiliations:** 1 Department of Physics and Materials Engineering, Faculty of Technology, Tomas Bata University in Zlín, Vavrečkova 275, 760 01 Zlín, Czech Republic; m1_kadleckova@utb.cz (M.K.); smolka@utb.cz (P.S.); mracek@utb.cz (A.M.); wrzecionko@utb.cz (E.W.); novak.libor7@seznam.cz (L.N.); lmusilova@utb.cz (L.M.); radek.gajda@seznam.cz (R.G.); 2 Centre of Polymer Systems, Tomas Bata University in Zlín, Třída Tomáše Bati 5678, 76001 Zlín, Czech Republic

**Keywords:** aluminum, alloy, duralumin, etching, surface texture, porous-like, adhesive bonding, superhydrophobic

## Abstract

The ways of producing porous-like textured surfaces with chemical etching on aluminum-alloy substrates were studied. The most appropriate etchants, their combination, temperature, and etching time period were explored. The influence of a specifically textured surface on adhesive joints’ strength or superhydrophobic properties was evaluated. The samples were examined with scanning electron microscopy, profilometry, atomic force microscopy, goniometry, and tensile testing. It was found that, with the multistep etching process, the substrate can be effectively modified and textured to the same morphology, regardless of the initial surface roughness. By selecting proper etchants and their sequence one can prepare new types of highly adhesive or even superhydrophobic surfaces.

## 1. Introduction

Aluminum alloys have long been used in many industrial applications, especially for their anticorrosion properties and low specific weight [1]. They have been utilized in construction, structural, cover, and front parts in the automotive, aviation, and astronautics industries, for the production of molds, etc. [2]. Especially for front and functional surfaces, which come into contact with other materials and weather conditions, aluminum alloys are essential postproduction processes for surface treatment [2,3,4]. These modifications should improve the appearance of the final product and inhibit surface oxidation [5,6,7], promote adhesion [8,9,10,11], or reduce staining thanks to their self-cleaning properties [4,12].

The composition of etchants for aluminum-alloy etching has been intensively studied [3,13,14,15,16,17,18,19,20,21]. The following chemicals are among those used: a hot sodium hydroxide solution with subsequent nitric acid application [3], phosphoric acid [13], acids with the addition of surfactants [19], salts [20], ferrous ions [14], mixtures of phosphoric and nitric acid with copper or ammonia ions [15], a mixture of hydrochloric acid with sulfuric acid or ethylene glycol [16], and hydrochloric acid alone [12,21]. 

Alloy composition and the surface-machining method also affect the course of the etching process and its result [17]. The etching rate can be controlled by temperature [3], and current density in the case of electrochemical etching [16,22,23].

The selected processes result in either smooth [13,23] or textured surfaces [4,12,18,21,22], which can exhibit hydrophobic properties when subsequent surface modification is applied [12,18,21,22]. Chemicals that are used for these modifications include fluorocarbons [12], stearic acid [5,6,18,24], polypropylene [25], and silanes [7,26].

In this work, the ways of producing porous-like textured surfaces on aluminum-alloy substrates with multistep etching were studied. The aim was to provide a method that allows preparing comparable final surfaces regardless of initial surface roughness and machining, with minimum material loss, and, from a practical point of view, to show how these new specifically textured surfaces influence adhesive bonding or wetting properties. 

## 2. Materials and Methods 

### 2.1. Materials

The studied material was an aluminum alloy (designed as duralumin, outlined further in the text) with a composition of 96.8 ± 0.1% Al, 2.6 ± 0.1% Mg, 0.5 ± 0.1% Fe, and others. The composition was determined with a delta element X-ray fluorescence spectrometer. Standard chemicals were purchased from Sigma-Aldrich (St. Louis, MO, USA) in p.a. purity. Ultrapure water with a resistivity of 18.2 MΩ·cm was used (Direct-Q ® 3UV, Merck, NJ, USA). Flexible polyurethane resin U4291 (ABchemie, Corbelin, France) was used for the preparation of adhesive bonds. 

### 2.2. Preparation of Duralumin Samples

Duralumin sheets of 1 mm thickness were used. Samples were cut into 2 cm × 6 cm and 2 cm × 1 cm pieces. The surface of the rolled sheets with an initial roughness of Ra ≤ 0.5 μm was modified by grinding or corundum blasting to a final roughness of Ra ≤ 3 μm and Ra > 6 μm, respectively. The sandpaper used for grinding was of a 180 grade, and the corundum particles had a mean size of about 90 μm. The mechanically treated samples were rinsed with acetone, water, and ethanol, and dried at 23 °C for 20 min prior to each experimental step. Prior to etching, the samples were conditioned to etching-bath temperature.

### 2.3. Etchant Composition

Etchants contained a base (NaOH) or a mixture of acids (37% HCl, 65% HNO_3_, 85% H_3_PO_4_, 96% H_2_SO_4_) with methanol (CH_3_OH) and sodium nitrite (NaNO_2_), and sodium nitrate (NaNO_3_). Etchants were prepared in glass beakers and conditioned to the desired temperature prior and during etching. 

### 2.4. Etching Process

The etching process was performed in glass beakers and the etchants were conditioned to a temperature in the range of 25 to 100 °C. Etching time was 1 to 10 min. The samples were rinsed with water and ethanol and allowed to dry at 23 °C between the individual etching-process steps. Afterward, the samples were kept in LDPE (low density polyethylene) bags in a desiccator. 

### 2.5. Preparation of Superhydrophobized Surfaces

The selected duralumin samples were also hydrophobized with stearic acid similarly to the literature [5,6,18,24]. First, the stearic acid was dissolved by stirring in an ethanol:water 1:1 weight fraction solution at 60 °C for 20 min. Then, the duralumin samples were introduced into the solution and left there for 29 h at 60 °C. Finally, the samples were removed and rinsed with ethanol and water. 

### 2.6. Preparation of Samples for Adhesion Testing

**Figure 1:**
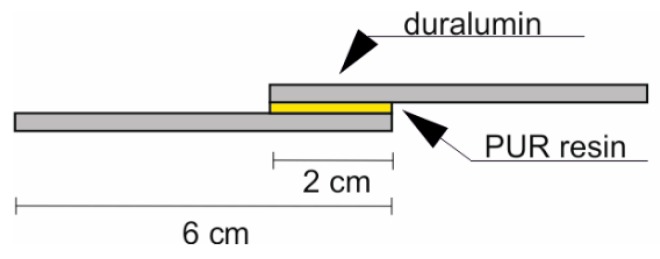
Sample for adhesion tests.

The 6 cm × 2 cm duralumin samples were joined with the U4291 polyurethane. The initial viscosity of the resin was 0.6 Pa·s for the material to reproduce the surface texture of the duralumin samples well. The samples were joined over the 2 cm × 2 cm area (Figure 1). Curing time was at least 72 h at 23 °C.

### 2.7. Scanning Electron Microscopy

Changes in surface appearance were analyzed by a Phenom Pro (Phenom-World BV, Eindhoven, The Netherlands) scanning electron microscope (SEM). The samples were observed at an acceleration voltage of 10 kV in backscattered electron mode.

### 2.8. Atomic Force Microscopy

Changes in the surface topography of the selected samples were characterized using a Ntegra-Prima (NT-MDT Spectrum Instruments, Moscow, Russia) atomic force microscope (AFM). Measurements were performed at a scan speed of 0.5 Hz with a resolution of 512 × 512 pixels, in tapping mode, at room temperature, in air atmosphere. A silicone-nitride probe with a resonant frequency of 150 ± 50 kHz and a stiffness constant of 5.5 N/m (NSG01, AppNano, Applied NanoStructures, Inc., Mountain View, CA, USA) was used. The data from the AFM measurement were processed in Gwyddion 2.5 software (Czech Metrology Institute, Jihlava, Czech Republic).

### 2.9. Profilometry

Changes in surface roughness (Ra) were characterized by a DiaVite DH-8 contact profilometer (Bülach, Switzerland). A diamond tip with a curvature radius of 2 microns was used. The evaluation of the surface roughness was performed according to the ASME B46.1 standard. Mean Ra values were determined from 15 individual measurements at various locations on three samples. Sample thickness was measured with a Mitutoyo 543-561D digital indicator (Kawasaki, Japan). 

### 2.10. Goniometry

The apparent and sliding contact angles of water on the stearic acid-modified duralumin surface were characterized with a drop shape analyzer DSA30, Krüss (Hamburg, Germany). Measurement was performed at 23 °C temperature. A drop with a volume of 3 µL (for the apparent contact angle) or 10 µL (for the sliding contact angle) was deposited on the measured surface. Ultrapure water with a resistance of 18.2 MΩ·cm was used for the measurement. All measurements were repeated 10 times; the mean values and standard deviations are presented in the results.

### 2.11. Adhesion Testing

The samples were prepared according to Section 2.6. Adhesion joint-strength evaluation was performed with an Instron 3345 universal testing machine (Norwood, MA, USA) with a 5 kN force sensor. Travel speed was 2 mm/min. The tests were performed in quintuplicate.

## 3. Results and Discussion

The first section of the results deals with the effect of etchant composition, temperature, and etching time on the duralumin surface topography. Based on these data, four etchants were chosen in order to prepare either a specifically structured surface or smooth surfaces. The last part is devoted to the effect of a porous-like surface structure on adhesive joint strength or wetting properties.

### 3.1. Etchant Composition

**Figure 2:**
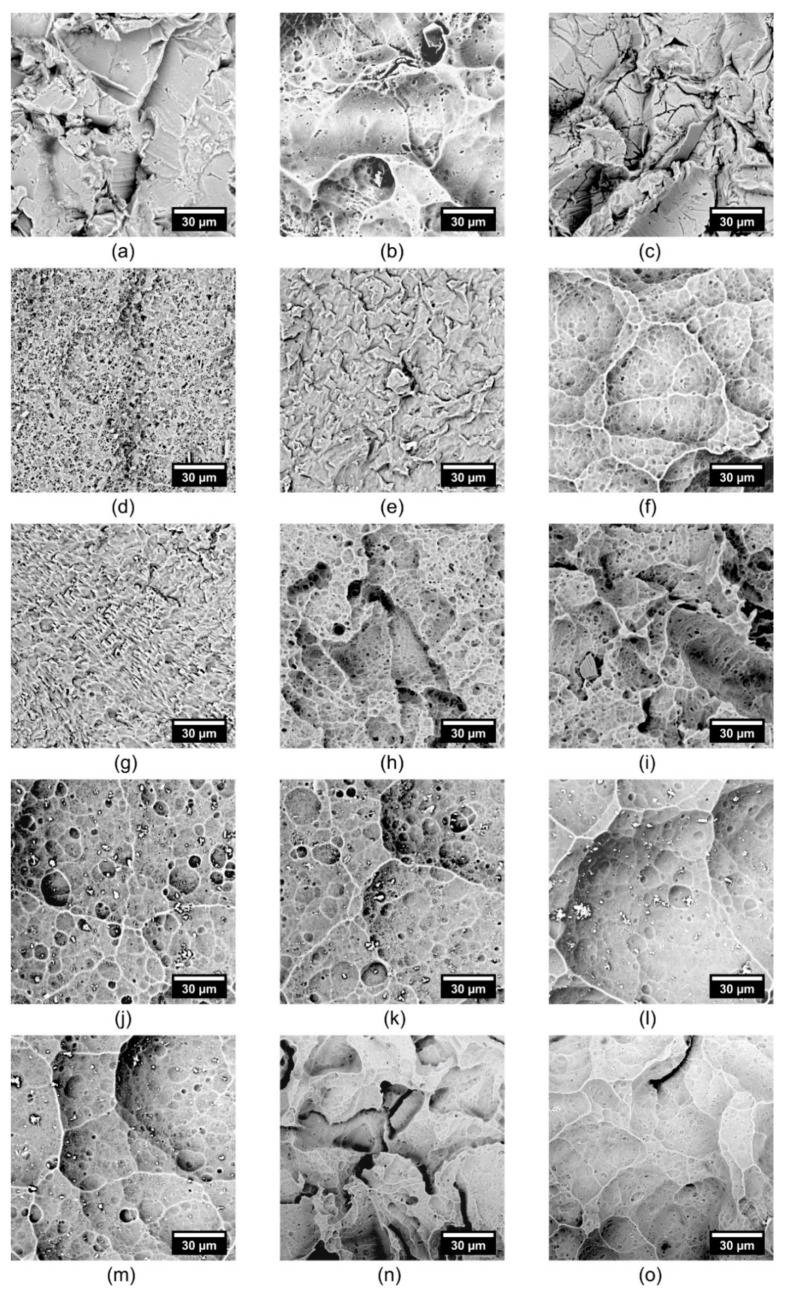
Scanning electron microscope (SEM) micrographs of etched duralumin surfaces. Designation corresponds with Table 1. Etching time 4 min, temperature 70 °C.

Etchant composition is vital for an effective etching process [3,13,14,15,16,17,18,19,20,21]. Various etchants were tested and optimized. Figure 2a–o shows the effect of etchant composition on surface relief and the roughness of the sandblasted duralumin. Etchant compositions, along with the achieved Ra values, are shown in Table 1. These data show that the most significant roughness reduction was achieved with the HNO_3_ + HCl or H_3_PO_4_ + HNO_3_ + HCl mixture, namely, from Ra 6.6 to 2.6 or 2.7 μm, respectively. In other cases, the effect was not so significant. 

Based on this preliminary testing and optimization, the following four etchants were chosen for further experiments:

- Etch Mix I (21 mL H_2_O + 9 g NaOH)
- Etch Mix II (21 mL H_3_PO_4_ + 3 mL HNO_3_ + 6 mL H_2_SO_4_)
- Etch Mix III (10 mL CH_3_OH + 10 mL HCl + 10 mL HNO_3_)
- Etch Mix IV (20 mL H_2_O + 9.8 mL HNO_3_+ 7.8 mL H_3_PO_4_ + 6 mL H_2_SO_4_ + 4 g NaNO_3_)

Etch Mix I for pre-etching and edge-chamfering, Etch Mix II for smooth surface, Etch Mix III for the generation of a specific texture, and Etch Mix IV for a porous-like pattern.

The choice of etchants was based on the comparison of data in Table 1 and Figure 2. Etch Mix I was chosen for its elimination of sharp edges in the sandblasted surface (Figure 2). The smoothing of surfaces can be observed in Figure 2o, Etch Mix II. The evolution of the specific surface relief and surface smoothing can be observed in Figure 2e, Etch Mix III. A porous-like pattern at a minimal change of the initial surface can be observed in Figure 2n, Etch Mix IV. The concentration of the components in individual etchants was modified so that the desired effect was achieved at the lowest possible temperature and time. In the case of the Etch Mix III, the experiments have shown that methanol can accelerate etching at a lower temperature. 

### 3.2. Temperature and Time Period

**Figure 3:**
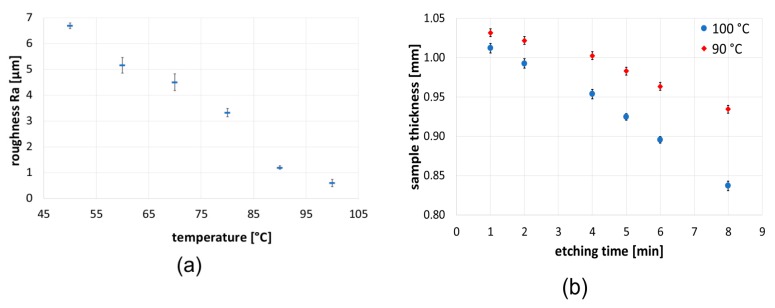
(**a**) Effect of temperature on surface roughness and (**b**) effect of etching time on sample-thickness reduction on sandblasted duralumin with Etch Mix I and Etch Mix II. The etching process with Etch Mix I was the same for both cases (5 min at 23 °C), the second step with Etch Mix II proceeded at either (**a**) a fixed time of 5 min and varying temperature or (**b**) a fixed temperature and varying etching time.

Important parameters affecting the etching process are the etchant temperature and etching time. A two-step process was utilized to demonstrate these effects (Figure 3). In the first step, the sample was etched for 5 min at 23 °C with Etch Mix I, and subsequently Etch Mix II for 5 min, either at various temperatures, or at one constant temperature and varying etching time. At 5 min etched time, the Ra value decreased with temperature, from 6.6 to 0.6 μm (Figure 3a). A similar trend can be observed in Figure 3b, where a given temperature decreased sample thickness with etching time. These observations correspond with the literature data for various etchants [3] and are essential from a practical point of view. Lowering the temperature from 100 °C to 90 °C results in a lower etching rate to such an extent that Ra reduction was no longer possible with some of the etchants. This fact demonstrates the dominating role of temperature in the etching process.

### 3.3. Initial Surface Roughness

It was found that the etching rate on sandblasted surfaces is much higher than on rolled or grinded (Table 2). This phenomenon was connected with a larger surface area and accelerated propagation of the etching process in the surface dents. The Ra value on grinded samples decreased from 2.6 ± 0.1 μm to 1.1 ± 0.1 μm, and thickness was reduced by 9%. In contrast, on the sandblasted samples the Ra value went from 6.6 ± 0.4 μm to 1.9 ± 0.2 μm with 25% thickness reduction. One can thus conclude that Etch Mix III was more aggressive on a surface with higher roughness. 

**Figure 4:**
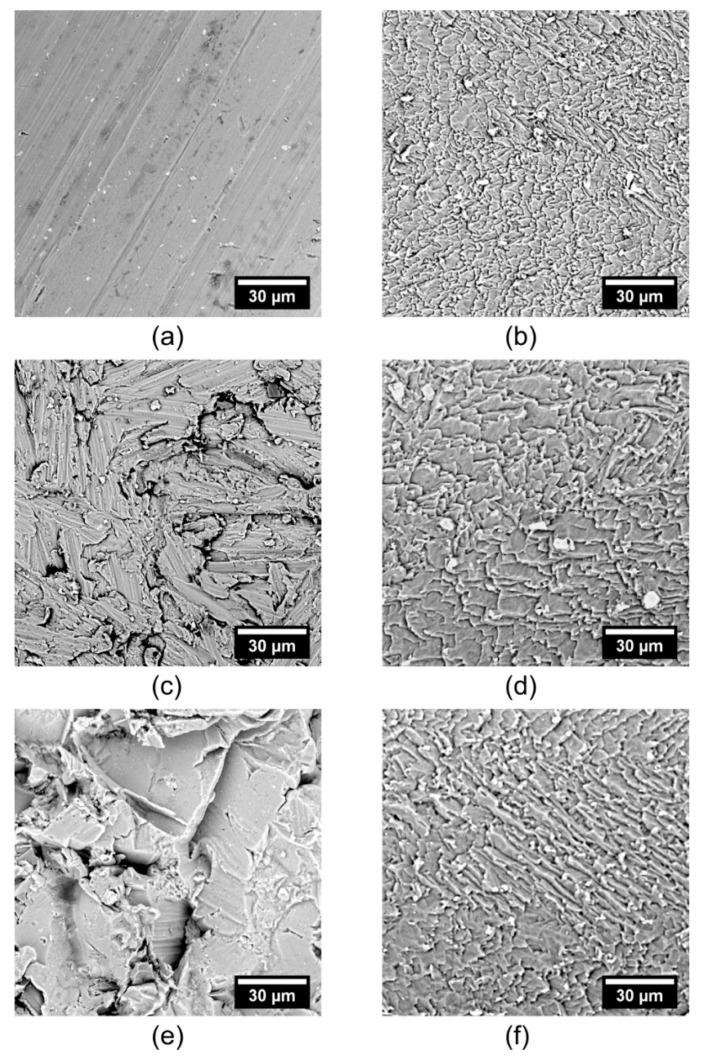
Effect of initial sample surface on the etching process with Etch Mix III at 23 °C for 3 min. Designation corresponds with the data in Table 2. (**a**) rolled surface; (**b**) etched rolled surface; (**c**) grinded surface; (**d**) etched grinded surface; (**e**) sandblasted surface; (**f**) etched sandblasted surface.

Figure 4 deals with the effect of the initial sample surface on the etching process with Etch Mix III at 23 °C for 3 min. Ra values and thickness data are shown in Table 2. The experiments revealed that a very similar surface can be prepared relatively rapidly at mild temperatures, regardless of texture of the initial surface. For comparison, see Figure 4b,d,f. Only on the smooth surface is the Ra value higher after etching compared to the initial surface. In the case of grinded and sandblasted surfaces, the trends are opposite and the Ra values decrease. In either case, all etched surfaces contain some kind of flakelike features, where the characteristic dimensions of the flakes are related to the texture of the initial surface, i.e., larger flakes were observed in the sample with a higher initial surface roughness, namely, Ra 0.7 ± 0.1, 1.1 ± 0.1, and 1.9 ± 0.2 µm in the treated rolled, ground, and sandblasted samples, respectively. For complete data, please refer to Table 2.

### 3.4. Preparation of Porous-Like Textured Surfaces

**Figure 5:**
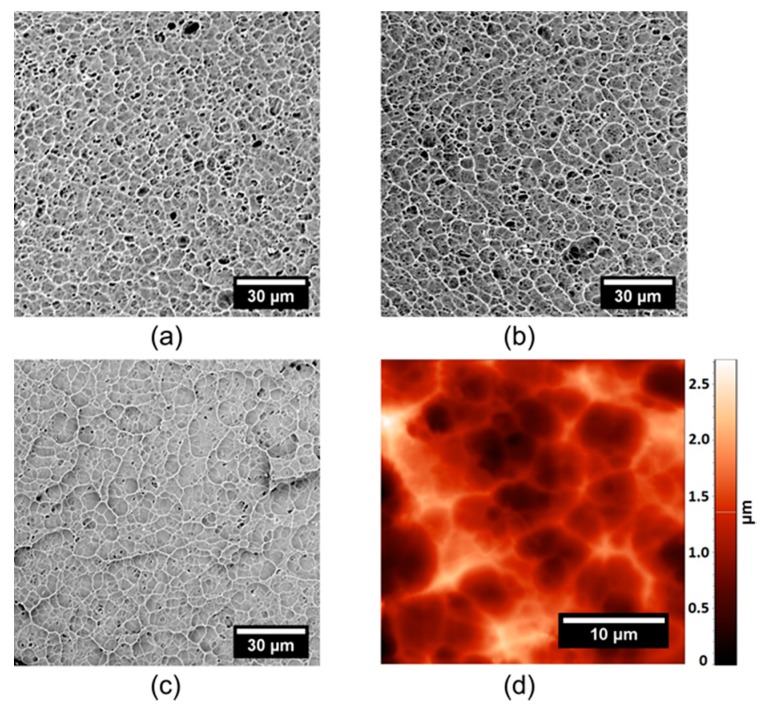
Porous-like surfaces prepared at (**a**) rolled, (**b**) grinded and (**c**,**d**) sandblasted duralumin. Surfaces etched with Etch Mix III and Etch Mix IV. (**a**–**c**) SEM and (**d**) atomic force microscope (AFM) micrographs.

To prepare porous-like surfaces, the samples in Figure 4b,d,f were exposed to further etching with Etch Mix IV at 80 °C for 3 min (see Figure 5). The Ra value increased at the rolled surface to 1.0 ± 0.1 µm and at the grinded surface to 0.9 ± 0.1 µm, and decreased at the sandblasted surface to 1.6 ± 0.2 µm. The surfaces thus had a similar appearance and roughness. The sandblasted samples were also analyzed with AFM (Figure 5d) and the area roughness parameter was determined, Sa = 0.4 µm.

When Etch Mix III and Etch Mix IV were applied in reverse order, i.e., first Etch Mix IV and then Etch Mix III, the surfaces were similar to those in Figure 4b,d,f. This means that the order of etchant application cannot be changed if we want to prepare porous-like surfaces. 

### 3.5. Preparation of Smooth Surface

**Figure 6:**
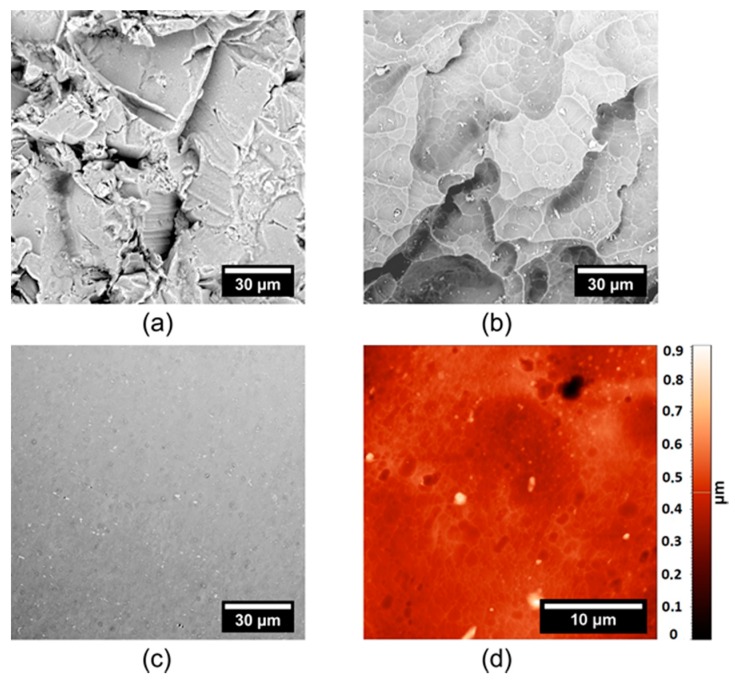
Flat surface prepared from sandblasted duralumin. (**a**) Initial surface, (**b**) etching with Etch-Mix-I, (**c**,**d**) etching with Etch-Mix-I and Etch-Mix-II. (**a**–**c**) SEM and (**d**) AFM micrographs.

The literature describes many ways for the preparation of a smooth duralumin surface [3]. Our experiments, presented in Figure 2 and Table 1, revealed that one-step etching at a mild temperature does not lead to surfaces with Ra under 1 µm. Thus, a multistep approach was developed, consisting of pre-etching with Etch Mix I at 23 °C for 5 min, with a subsequent application of Etch Mix II at 100 °C for 5 min (Figure 6). In the first step, the edges or surface microstructures are chamfered, and the Ra slightly decreases from 6.6 ± 0.4 µm (Figure 6a) to 6.2 ± 0.1 µm (Figure 6b). In the second step. the Ra value drops dramatically to 0.6 ± 0.1 μm (Figure 6c). The final surface was also analyzed with AFM, and the area roughness parameter was Sa = 0.08 µm. 

Further text demonstrates that the prepared surfaces can be used for many distinct applications.

### 3.6. Adhesive Bonding of Textured Surfaces

The strength of the adhesive joints is determined by chemical composition of substrates and their surface texture. It is known that textured surfaces influence material-utility properties, increase adhesion [8,9,10,11], and implicate the development of cell systems [27,28]. 

**Figure 7:**
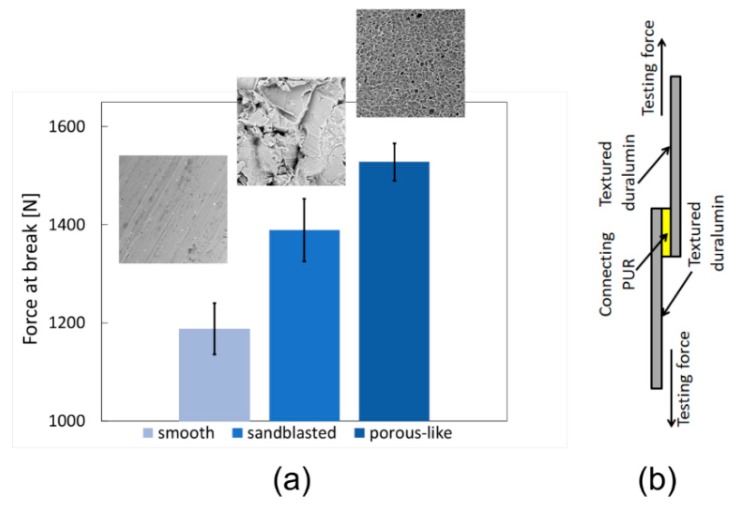
(**a**) Adhesive joint strength according to substrate surface texture. (**b**) Experimental setup.

Prepared porous-like (Figure 5) and smooth (Figure 6) surfaces were tested by means of adhesive joint strength (Figure 7a). The overlapping 2 cm × 2 cm area was strained according to Figure 7b. The following forces at break were recorded: 1188 N for smooth duralumin, 1390 N for sandblasted duralumin, and 1528 N for porous-like duralumin (Figure 7a). These data indicate that periodic surface texture can be more beneficial in increasing adhesion strength than a high Ra value—porous-like surface vs. sandblasted surfaces. There is a limit to this statement, as seen in the adhesion data for smooth surface with an Ra equal 0.6 µm, which had the worst adhesion strength. 

### 3.7. Wetting Properties

**Figure 8:**
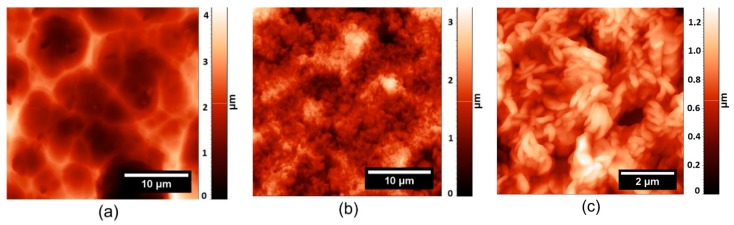
AFM micrographs of a porous-like duralumin surface (**a**) after chrome sulfuric acid application, and (**b**,**c**) stearic acid application.

The literature suggests that it is possible to increase duralumin’s hydrophobic properties with stearic acid coating [6,18,24]. However, none of the described approaches was as efficient as our procedure. It was found that the most important step is the exposition of duralumin to chrome sulfuric acid for 2 min at 23 °C to remove surface oxides and etching-process residues. The process is followed by boiling the samples in water for 5 min. Then, the samples were exposed to stearic acid, as described in Section 2.5. With this approach, surfaces with an apparent water contact angle of almost 170° and a sliding angle of about 4° can be achieved, which is the highest reported value for stearic acid modification [5,6,18]. As shown in Figure 8b,c, stearic acid forms a nanotexture on the porous-like surface, thus contributing to the hydrophobic properties. Superhydrophobic surfaces feature a combination of a specific chemical composition along with specific surface micro- and nanotexture [4,12,18,21,22]. Without the chrome sulfuric acid treatment, only apparent water contact angles of about 150° can be achieved. The apparent water contact angles of stearic acid-treated surfaces are presented in Table 3. 

## 4. Conclusions

This work deals with the combination of different etching mixtures, their composition, temperature, and etching time on the modification of aluminum-alloy substrates in order to prepare either porous-like structured or smooth surfaces. It was found that the appropriate combination of etchants applied in a precise order can be used to prepare porous-like textured surfaces with an Ra of 1.6 µm or smooth substrates with an Ra below 0.8 µm. Such modifications are possible regardless of initial surface roughness or surface machining. 

In order to prepare a specific surface microtexture, it is convenient to especially combine the mixture of nitric and hydrochloric acids with methanol. The methanol addition significantly promotes the etching process at rough surfaces and allows temperature reduction from 80 °C to 23 °C. 

From a practical point of view, it was demonstrated that the porous-like surfaces can either promote adhesive bonding or allow the effective preparation of superhydrophobic surfaces featuring a self-cleaning effect due to high apparent water contact angles. 

## Figures and Tables

**Table 1:** Etchant composition and resultant surface roughness. Sandblasted duralumin etched at 70 °C for 4 min.

Label	Etching Mixture	Mixture Ration	Ra (µm)
(a)	None (sandblasted surface)	-	6.6 ± 0.4
(b)	H_3_PO_4_	10 mL	6.7 ± 0.7
(c)	HNO_3_	10 mL	5.2 ± 0.6
(d)	H_3_PO_4_ + HCl	5:5 mL	4.7 ± 0.9
(e)	HNO_3_ + HCl	5:5 mL	2.6 ± 0.8
(f)	H_3_PO_4_ + HNO_3_	5:5 mL	7.5 ± 2.2
(g)	H_3_PO_4_ + HNO_3_ + HCl	3.5:3.5:3.5 mL	2.7 ± 0.5
(h)	H_3_PO_4_ + HNO_3_ + HCl + H_2_SO_4_	2.5:2.5:2.5:1 mL	5.3 ± 0.8
(i)	H_3_PO_4_ + HNO_3_ + HCl + H_2_SO_4_ + H_2_O	2.5:2.5:2.5:1:1 mL	5.6 ± 0.7
(j)	H_2_O + NaOH	10 mL:0.4 g	6.0 ± 2.4
(k)	H_2_O + NaOH + NaNO_2_	10 mL:0.4 g:8 g	4.9 ± 0.8
(l)	H_2_O + NaOH + NaNO_3_	10 mL:0.4 g:2 g	7.5 ± 0.7
(m)	H_2_O + NaOH + NaNO_2_ + NaNO_3_	10 mL:0.4 g:8 g:2 g	6.8 ± 1.5
(n)	H_2_O + HNO_3_ + H_3_PO_4_ + H_2_SO_4_ + NaNO_3_	10 mL:9.8 mL:7.8 mL:6 mL:4 g	6.0 ± 0.4
(o)	HNO_3_ + H_3_PO_4_ + H_2_SO_4_	3.5:3.5:3.5 mL	5.2 ± 0.2

**Table 2:** Roughness and thickness reduction in samples with various initial surface characteristics. Etched with Etch Mix III for 3 min at 23 °C.

Label	Sample	Thickness (mm)	Ra (µm)
(a)	Rolled surface	1.08 ± 0.01	0.3 ± 0.1
(b)	Etched rolled surface	1.05 ± 0.01	0.7 ± 0.1
(c)	Grinded surface	1.09 ± 0.01	2.6 ± 0.1
(d)	Etched grinded surface	1.00 ± 0.01	1.1 ± 0.1
(e)	Sandblasted surface	1.09 ± 0.01	6.6 ± 0.4
(f)	Etched sandblasted surface	0.82 ± 0.01	1.9 ± 0.2

**Table 3:** Water contact angles of duralumin surfaces with applied stearic acid

Surface	Water Contact Angle (°)		Ra before Stearic Acid (µm)
	Apparent	Sliding	
Rolled	155 ± 1	15 ± 3	0.3 ± 0.1
Grinded	157 ± 2	12 ± 3	2.6 ± 0.1
Sandblasted	165 ± 2	10 ± 2	6.6 ± 0.4
Porous-like	169 ± 1	4 ± 1	1.6 ± 0.2
